# Antiproteinuric Response to Finerenone in Diabetic Kidney Disease: Responder and Non-Responder Subgroup Analysis of the FINDKDLATAM Study

**DOI:** 10.3390/clinpract16100174

**Published:** 2026-09-22

**Authors:** Jorge Rico Fontalvo, María Raad Sarabia, José C. De La Flor, Vicente Sánchez Polo, Jenniffer Benavides Garcia, Enrique Ramos Clason, Daniel Domínguez, Giovanny Mera Rebutti, Carlos Madrid Mancia, Rene Tabora López, Manuel Rocha Meza, Dany Tabora López, James J. Muñoz Zambrano, Eduardo Lorca Herrera, Eliana Dina-Batlle, Michael Cieza Terrones, Alvaro Pedroza Pallares, Tomas Rodríguez Yánez, Washington Osorio, Alyi Arellano Cabeza, Juan Felipe Gutiérrez, Avinash Chandu Nanwani, Celia Rodríguez Tudero, Rodrigo Daza Arnedo

**Affiliations:** 1 Latin American Society of Nephrology and Hypertension (SLANH), Panamá City 0801, Panama; jorgericof@yahoo.com (J.R.F.); visanpolo@gmail.com (V.S.P.); drdomingueznefrologia@gmail.com (D.D.); manuelrochameza@yahoo.com (M.R.M.); edulorcah@gmail.com (E.L.H.); dradina@me.com (E.D.-B.); rodrigoandres_2@hotmail.com (R.D.A.); 2 Colombian Association of Nephrology and Arterial Hypertension, Bogota 110221, Colombia; dr.alvaro.pedrozapallares@gmail.com (A.P.P.); juanfe324@gmail.com (J.F.G.); 3 Department of Nephrology, Faculty of Medicine, Simón Bolívar University of Barranquilla, Barranquilla 080001, Colombia; 4 Department of Nephrology, Hospital Central de la Defensa Gómez Ulla, 28047 Madrid, Spain; 5 Department of Medicine and Medical Specialties, Faculty of Medicine, Alcala University, 28805 Madrid, Spain; 6 Faculty of Medicine, Department of Nephrology, University of San Carlos of Guatemala (USAC), Guatemala City 01011, Guatemala; 7 Department of Epidemiology, El Rosario University, Bogota 111221, Colombia; jennben89@gmail.com; 8 Medical and Surgical Research, GIBACUS Research Group, Sinú University EBZ, Cartagena Campus, Cartagena 130010, Colombia; drramosclason@gmail.com (E.R.C.); trodriguezy123@gmail.com (T.R.Y.); 9 Faculty of Nursing, Health Services Administration Program, University of Cartagena, Cartagena 130005, Colombia; 10 Department of Nephrology, Del Valle Hospital, Catholic University of Honduras, San Pedro Sula 21102, Honduras; drtaboralopezmi@gmail.com (R.T.L.); dannytabora29@gmail.com (D.T.L.); 11 Department of Nephrology, Dr. Abel Gilbert Pontón Hospital of Guayaquil, Ministry of Public Health, O’Connors, Guayaquil 090104, Ecuador; gomerarebutti@gmail.com; 12 Department of Nephrology, Honduran Institute of Social Security, North Region, San Pedro Sula 21102, Honduras; drmadrid83@yahoo.com; 13 Department of Nephrology, Bendaña Hospital, San Pedro Sula 21102, Honduras; 14 Internal Medicine, Mario Catarino Rivas Hospital, San Pedro Sula 21102, Honduras; 15 Department of Nephrology, Rafael Rodríguez Zambrano Hospital, Manta 130206, Ecuador; drjamesmunoz@hotmail.com; 16 Department of Internal Medicine East, Faculty of Medicine, University of Chile, Providencia, Santiago 8320000, Chile; 17 Department of Nephrology, Metropolitan Hospital of Santiago, Santiago de los Caballeros 51000, Dominican Republic; 18 Department of Engineering, Faculty of Science and Engineering, Peruana Cayetano Heredia University, Lima 15012, Peru; michael.cieza@upch.pe; 19 Department of Nephrology, San Jose Maicao ESE Hospital, Maicao 442001, Colombia; 20 Department of Internal Medicine, Faculty of Medicine, University of Cartagena, Cartagena 130005, Colombia; 21 Department of Nephrology, Armed Forces Specialty Hospital Number 1, Quito City 170403, Ecuador; dr.osoriow@yahoo.com; 22 Department of Internal Medicine, University of Valle, Cali 760042, Colombia; alyi60428@gmail.com; 23 Department of Nephrology, Davita Kidney Care, Barranquilla 080020, Colombia; 24 Department of Nephrology, Hospital General de Fuerteventura, 35600 Puerto del Rosario, Spain; achanan@gobiernodecanarias.org; 25 Department of Nephrology, Hospital Señora de Sonsoles, 05004 Avila, Spain; crodrigueztudero@usal.es; 26 Surgery Department, Faculty of Medicine, University of Salamanca, 37007 Salamanca, Spain

**Keywords:** finerenone, diabetic kidney disease, responder, albuminuria, Latin America

## Abstract

**Background/Objectives**: Albuminuria is an early marker of kidney damage and an independent predictor of adverse outcomes, making its reduction a key therapeutic goal. Finerenone, a selective nonsteroidal mineralocorticoid receptor antagonist, has demonstrated renal and cardiovascular benefits in patients with diabetic kidney disease (DKD), although the magnitude of the antiproteinuric response may vary in clinical practice. This post hoc analysis aimed to characterize the six-month antiproteinuric response to finerenone and to descriptively explore baseline clinical and treatment characteristics across response categories in the FINDKDLATAM cohort. **Methods**: A subgroup analysis was performed on a real-world, multicenter, retrospective, observational study that included patients from the FINDKDLATAM study who had urinary albumin-to-creatinine ratio (UACR) measurements taken at baseline and six months. Patients were classified as absolute responders (≥50% reduction in UACR), partial responders (30–49% reduction), and non-responders (<30% reduction or no reduction in UACR) at six months of follow-up. Sociodemographic, clinical, biochemical, therapeutic, and safety variables were analyzed. **Results**: Of the 347 patients included in the original cohort, 334 had complete UACR data at six months and were included in the analysis. Of these, 80.2% (*n* = 268) were absolute responders, 11.7% (*n* = 39) partial responders, and 8.1% (*n* = 27) non-responders. Median UACR decreased from 350.7 to 67.0 mg/g among absolute responders and from 240.5 to 143.0 mg/g among partial responders (both *p* < 0.0001), whereas no significant change was observed among non-responders. No baseline clinical characteristic consistently distinguished responders from non-responders. Serum potassium increased during follow-up, particularly among non-responders. **Conclusions**: In this retrospective Latin American cohort, most patients experienced a reduction in UACR after six months of finerenone treatment. However, the uncontrolled observational design, concomitant therapies, UACR variability, and regression to the mean preclude attributing these changes exclusively to finerenone. These exploratory findings do not support selecting patients according to a particular responder phenotype but rather support prescribing finerenone according to currently approved clinical criteria. Prospective studies with adjusted analyses are needed to identify independent predictors of response.

## 1. Introduction

Diabetic kidney disease (DKD) is the leading cause of chronic kidney disease (CKD) worldwide and constitutes one of the most relevant microvascular complications of type 2 diabetes mellitus (T2DM) [1]. Its presence is associated with a greater probability of progression to end-stage kidney disease, the need for kidney replacement therapy and a relevant increase in cardiovascular risk and mortality. The increasing global prevalence of T2DM, triggered by population aging, obesity and changes in lifestyle, has made DKD a priority public health problem [1]. In addition to the metabolic alterations inherent to diabetes, mechanisms such as chronic low-grade inflammation, oxidative stress, neurohormonal activation and fibrosis contribute to progressive kidney damage and the development of cardiovascular–kidney–metabolic (CKM) syndrome [1].

Albuminuria constitutes one of the most sensitive and clinically useful markers for the early identification of kidney damage associated with diabetes [2,3]. Its presence reflects alterations in the glomerular filtration barrier and is correlated with a greater risk of deterioration of the kidney function, cardiovascular events and mortality [4,5]. Currently, both the urinary albumin-to-creatinine ratio (UACR) and the estimated glomerular filtration rate (eGFR) are the pillars for the diagnosis, classification and follow-up of CKD [6]. Several studies have proved that the sustained reduction in albuminuria is associated with slower progression of kidney disease and a decrease in cardiovascular risk, which is why it is considered a main therapeutic objective in patients with DKD [4,5].

In recent years, the treatment of DKD has undergone a significant transformation with the inclusion of therapies that are able to modify the natural history of the disease [5,6,7]. Sodium-glucose cotransporter 2 (SGLT-2) inhibitors have shown a consistent reduction in kidney disease progression and cardiovascular events in patients with T2DM and CKD, independently of their glycemic effect [7]. These benefits seem to be related to hemodynamic, metabolic and anti-inflammatory mechanisms that contribute to reducing glomerular hyperfiltration and progressive kidney injury.

Finerenone, a selective nonsteroidal mineralocorticoid receptor antagonist (MRA), was developed to block pathogenic pathways mediated by the activation of the MR, with a more favorable safety profile than traditional steroidal antagonists [8]. The FIDELIO-DKD and FIGARO-DKD studies have shown that finerenone significantly reduces the progression of kidney disease and cardiovascular events in patients with T2DM and CKD treated with standard therapy based on renin–angiotensin system inhibition [9,10]. Later on, the FIDELITY program confirmed these benefits in a wide spectrum of patients, establishing finerenone as a main tool within the modern strategy of cardiorenal protection [11].

Although randomized trials have established the kidney and cardiovascular benefits of finerenone, substantial interindividual variability in albuminuria reduction has been observed with kidney-protective therapies. This variability is clinically relevant because early changes in UACR are associated with subsequent kidney outcomes. An individual participant-level meta-analysis showed that a 30% reduction in UACR was associated with a lower subsequent risk of end-stage kidney disease, while a meta-analysis of randomized trials supported an approximately 30% reduction in geometric mean UACR as a reasonably likely indicator of long-term kidney benefit [12,13,14]. Nevertheless, an early reduction in UACR remains an intermediate response marker and should not be interpreted as equivalent to a demonstrated reduction in hard kidney or cardiovascular outcomes.

The original FINDKDLATAM study evaluated the overall effectiveness and safety of finerenone in a Latin American real-world cohort, primarily reporting cohort-level changes in UACR, eGFR, blood pressure, and serum potassium [15]. However, it did not examine the distribution of individual UACR responses or assess whether patients with different magnitudes of response had distinguishable baseline clinical or treatment characteristics. Consequently, it remains uncertain whether the overall reduction observed in the original cohort was broadly distributed or predominantly driven by a subgroup of patients with a marked antiproteinuric response.

To address this gap, the present post hoc analysis classified patients according to the magnitude of their six-month UACR reduction. A reduction of at least 30% was selected as the threshold for a clinically meaningful response based on its reported association with a lower risk of kidney disease progression [13,14]. Within this group, a reduction of at least 50% was used as an exploratory threshold to identify patients with a more pronounced response, rather than as a universally validated clinical cutoff. Accordingly, patients were categorized as absolute responders (≥50% reduction in UACR), partial responders (30–49% reduction), and non-responders (<30% reduction or no reduction in UACR).

This analysis aimed to characterize the distribution of the antiproteinuric response to finerenone and to descriptively explore baseline clinical and treatment characteristics across response categories. By shifting from the cohort in the original FINDKDLATAM study to patient-level response categories, this analysis aimed to describe the heterogeneity of six-month UACR changes. It was not designed to identify independent predictors of response or to estimate the treatment-specific effect of finerenone.

## 2. Materials and Methods

This study was a post hoc analysis of the FINDKDLATAM (Finerenone in Diabetic Kidney Disease in Latin America) cohort, a multicenter, retrospective observational study of patients with diabetic kidney disease who initiated finerenone as part of routine clinical care across seven Latin American countries [15]. The present analysis aimed to characterize the distribution of six-month UACR changes and to descriptively explore baseline clinical and treatment characteristics across the predefined response categories. The original study included patients with T2DM and CKD treated with finerenone in specialized nephrology centers in Peru, Argentina, Mexico, Panama, Guatemala, Dominican Republic and Ecuador. The response categories were defined specifically for the present analysis and were not prespecified in the protocol of the parent study.

For the current analysis, patients in the original cohort who had UACR measurements at treatment initiation and at six months of treatment were included. Patients without complete albuminuria data during follow-up were excluded from the analysis.

The antiproteinuric response to finerenone was evaluated as the percentage change in UACR between the baseline value and the measurement obtained at six months of follow-up, using the following formula:

Percentage UACR change: (baseline UACR − six-month UACR)/(baseline UACR) * 100

Patients were classified into three categories: absolute responders: a decrease of ≥50% in UACR relative to the baseline value; partial responders: a reduction between 30% and 49% of the UACR; and non-responders: a reduction of <30% in UACR or no decrease in albuminuria. This classification was established with the purpose of identifying clinical characteristics associated with the magnitude of the antiproteinuric response observed during the treatment with finerenone. A reduction of at least 30% was considered a clinically meaningful response based on previous studies linking changes in this magnitude to a lower risk of kidney disease progression [13,14]. The ≥50% threshold was used as an exploratory cutoff to distinguish patients with a more pronounced reduction in UACR and should not be interpreted as a universally validated response threshold.

Demographic, clinical, biochemical and therapeutic variables obtained from the electronic medical records were collected. The demographic variables included age and gender. Among the clinical variables, history of arterial hypertension (HTN), atherosclerotic cardiovascular disease (ASCVD), heart failure (HF), obesity and T2DM duration were recorded. The biochemical variables included serum creatinine and eGFR calculated through the CKD-EPI 2021 equation, serum potassium concentration and UACR. In addition, the concomitant use of nephroprotective therapies, including SGLT-2 inhibitors, glucagon-like peptide-1 receptor (GLP-1R) agonists and the renin–angiotensin–aldosterone system (RAAS) inhibitors, was documented.

The primary outcome was the proportion of patients who achieved an absolute or partial antiproteinuric response at six months of treatment with finerenone. Secondary outcomes included changes in UACR, eGFR, systolic and diastolic blood pressure (SBP, DBP) and serum potassium levels, with the latter being a treatment-related adverse event.

Patients were followed for six months from the initiation of finerenone therapy. The baseline and final values of the clinical and biochemical variables available during the usual care were registered. Finerenone doses were determined by the treating physician, according to current recommendations and the clinical characteristics of each patient.

Baseline and six-month UACR values were obtained retrospectively from electronic medical records and corresponded to single measurements performed as part of routine clinical care. Baseline UACR was defined as the measurement closest to, and obtained before or at, finerenone initiation, whereas follow-up UACR was defined as the measurement closest to six months after treatment initiation within an allowable window of five to seven months. Repeated UACR measurements were not systematically available; therefore, the values could not be averaged or confirmed using a second sample. Given the known intraindividual biological and analytical variability of UACR, patients with changes close to the response thresholds may have been misclassified.

For the statistical analysis, the database was compiled in Microsoft Excel and subsequently analyzed using SPSS software version 30. Continuous variables were assessed for distributional assumptions using graphical methods and the Shapiro–Wilk test. Normally distributed variables are presented as mean and standard deviation, whereas non-normally distributed variables are presented as median and interquartile range. Categorical variables are reported as frequencies and percentages. Intragroup changes between baseline and follow-up measurements were evaluated using paired-samples tests, according to the distribution of the variables. Baseline characteristics across the three response categories were summarized descriptively, and no formal inferential comparisons were performed between categories. Within each response category, changes in continuous variables from baseline to six months were evaluated using the paired Student’s *t*-test or the Wilcoxon signed-rank test, according to the distribution of the data. These within-group analyses were not interpreted as evidence of differences in the magnitude of change between response categories. All tests were two-sided, and a *p*-value <0.05 was considered statistically significant.

No multivariable regression analysis was performed. The post hoc nature of the analysis, the small number of patients across some categories and the sparse distribution of patients across some treatment categories limited the number of parameters that could be estimated reliably. A model including the clinically relevant baseline and treatment variables would have been at substantial risk of overfitting and unstable estimates. Therefore, comparisons across response categories were considered unadjusted and exploratory and were not used to identify independent predictors of response.

Finally, regarding ethical aspects, the original FINDKDLATAM study [16] was conducted in accordance with the main ethical principles of the Helsinki Declaration and the local laws of each participating country. Due to its observational and retrospective nature, the information was obtained from previously existing clinical registries and was handled anonymously in order to guarantee the confidentiality of the patients.

## 3. Results

The original FINDKDLATAM study [16] included a total of 347 patients. During follow-up, 13 cases were excluded due to the absence of UACR data at six months; therefore, the subgroup analysis was finally performed on 334 patients. At six months, 80.2% of the patients (*n* = 268) were classified as absolute responders, 11.7% (*n* = 39) as partial responders and 8.1% (*n* = 27) as non-responders. Given the marked imbalance in group sizes, particularly the small number of non-responders, comparisons across response categories were considered exploratory and estimates involving the non-responder group should be interpreted with caution.

The group of absolute responders was made up of 268 patients (80.2%). The mean age was 63.4 ± 11.8 years and 45.9% were women. At the beginning of the study, the median UACR was 350.7 mg/g (interquartile range, IQR: 200–780), the eGFR was 37.6 mL/min/1.73 m^2^ (IQR: 30–48), the SBP was 145 mmHg (IQR: 133–160) and the serum potassium was 4.4 mEq/L (IQR: 4.1–4.6). At six months of follow-up, the UACR decreased to 67 mg/g (IQR: 27–128), which represented a decrease of 81.4% in relation to the baseline value (*p* < 0.0001) (Figure 1). The eGFR remained stable, with a median of 40.77 mL/min/1.73 m^2^ (*p* = 0.9714), while SBP decreased significantly to 130 mmHg (IQR: 120–140) (*p* < 0.0001). The serum potassium increased slightly up to 4.7 mEq/L (IQR 4.38–5.0) (*p* < 0.0001) (Table 1).

Thirty-nine patients (11.7%) were classified as partial responders. The mean age was 61.7 ± 11.9 years and 43.6% were women. Baseline values showed a median UACR of 240.5 mg/g (IQR: 73.66–347.75), eGFR of 46.15 mL/min/1.73 m^2^ (IQR: 40.25–56.0), SBP of 135 mmHg (IQR: 130–146.5) and serum potassium of 4.35 mEq/L (IQR: 3.9–4.5). After six months of treatment, the UACR decreased to 143.0 mg/g (IQR: 37.3–205.5), equivalent to a reduction of 40.5% in relation to the baseline value (*p* < 0.0001) (Figure 1). The eGFR remained stable at 48.8 mL/min/1.73 m^2^ (*p* = 0.0831). A significant reduction in SBP was also observed to 130 mmHg (IQR: 122.5–139.5) (*p* < 0.0001). Serum potassium showed a slight increase to 4.4 mEq/L (IQR: 4.15–4.75) (*p* = 0.0045).

The group of non-responders included 27 patients (8.1%). These patients had a mean age of 58.0 ± 10.8 years and 40.7% were women. At the beginning of the study, the median UACR was 314.5 mg/g (IQR: 190–489.5), the eGFR was 41.0 mL/min/1.73 m^2^ (IQR 33–53), the SBP was 147 mmHg (IQR: 120–170) and the serum potassium was 4.6 mEq/L (IQR: 4.43–4.98). At six months of follow-up, no significant changes in albuminuria were observed, with a UACR of 336 mg/g (IQR 197.75–693.75), representing an approximate increase of 6.8% in relation to the baseline value (*p* = 0.4434) (Figure 1). These patients presented a decrease in eGFR to 37 mL/min/1.73 m^2^ (IQR: 20–51) (*p* = 0.0023). The reduction in SBP did not reach statistical significance (*p* = 0.0592), while the serum potassium increased up to 5.15 mEq/L (IQR: 4.72–5.58) (*p* = 0.0006).

Points represent median UACR values, and bars represent interquartile ranges. Lines connect the summary values at both time points within each response category and do not represent individual patient trajectories. UACR: urinary albumin-to-creatinine ratio.

Response categories were descriptively distributed according to the concomitant treatment regimen. Among patients receiving finerenone plus an SGLT2 inhibitor, 74 of 75 (98.7%) were absolute responders, none were partial responders, and one (1.3%) was a non-responder. Among those receiving finerenone plus a RAAS inhibitor and an SGLT2 inhibitor, 113 of 150 (75.3%) were absolute responders, 22 (14.7%) were partial responders, and 15 (10.0%) were non-responders. In the group receiving finerenone plus a RAAS inhibitor, an SGLT2 inhibitor, and a GLP-1 receptor agonist, 10 of 16 (62.5%) were absolute responders, three (18.8%) were partial responders, and three (18.8%) were non-responders. Among patients receiving other regimens, 71 of 93 (76.3%) were absolute responders, 14 (15.1%) were partial responders, and eight (8.6%) were non-responders (Table 2). These unadjusted descriptive distributions should not be interpreted as evidence of comparative treatment effectiveness because treatment allocation was not randomized and baseline differences among treatment groups were not formally assessed.

## 4. Discussion

In this post hoc analysis of the FINDKDLATAM Latin American cohort [15], patients treated with finerenone showed a substantial reduction in UACR over six months. Overall, 91.9% of patients had a UACR reduction of at least 30% at six months, including 80.2% with a reduction of at least 50%. These proportions describe within-patient UACR changes among individuals treated with finerenone who had measurements available at both time points. However, they should not be interpreted as the proportion of responses caused by finerenone because the study did not include a concurrent control group. These findings are particularly relevant because the early reduction in albuminuria is currently considered a validated surrogate marker of long-term kidney protection and is associated with slower progression to end-stage kidney failure and a reduction in cardiovascular risk [2,4], which has led to its current incorporation in clinical practice guidelines as a therapeutic target [16].

The observed UACR changes should be interpreted separately from the treatment effects reported in FIDELIO-DKD and FIGARO-DKD [9,10]. Those randomized trials evaluated changes in UACR relative to placebo, whereas the present analysis describes within-patient changes from baseline without a comparator group. In addition, the proportion of patients exceeding a specified reduction threshold is a different measure from an average placebo-adjusted treatment effect.

Randomized trials estimate the average treatment effect relative to placebo, whereas the present analysis classified individual patients according to the percentage change from their own baseline UACR. Furthermore, the study population consisted of patients selected to receive finerenone in routine nephrology practice, many of whom were also receiving other kidney-protective therapies. Differences in patient selection, baseline UACR, concomitant treatment, clinical monitoring, and availability of follow-up measurements may therefore have influenced the observed response distribution. These estimates are therefore not directly comparable, and the present findings should not be interpreted as evidence of a greater antiproteinuric effect than that observed in randomized trials.

Several factors other than finerenone may have contributed to the observed changes in UACR. These include changes in blood pressure and glycemic control, concomitant RAAS inhibition, treatment with SGLT2 inhibitors or GLP-1 receptor agonists, and other therapeutic interventions implemented during follow-up. In addition, UACR has substantial intraindividual biological and analytical variability. Because response classification was based on a single measurement at baseline and another at six months, random variation and regression to the mean may have influenced both the magnitude of the observed reductions and the assignment of patients to response categories. Misclassification is particularly possible among patients with changes close to the 30% and 50% thresholds.

A finding of special interest was the difference observed in the evolution of kidney function according to the degree of antiproteinuric response. While the eGFR remained stable in the absolute and partial responders, patients classified as non-responders experienced a decline in kidney function during follow-up. However, no direct statistical comparison of eGFR change between categories was performed, and the separate within-group analyses do not establish that kidney function trajectories differed among them. Therefore, the observed eGFR values cannot be interpreted as evidence that UACR reduction was associated with preservation of kidney function. Interpretation is further limited by the small number of non-responders, the short follow-up period, potential baseline differences, and the absence of adjustment for confounding factors. Prospective studies with longer follow-up and direct between-group comparisons are needed to determine whether the magnitude of early UACR reduction is associated with subsequent changes in kidney function [5,17,18].

In addition to the changes observed in the albuminuria, the absolute and partial responders presented a significant SBP reduction during follow-up. This finding is consistent with the mechanisms of action described for finerenone and its capacity to modulate MR activation, decreasing vascular inflammation, arterial stiffness and sodium retention [8]. The absence of a significant reduction in blood pressure in the non-responders could reflect pathophysiological differences that deserve to be explored in future studies.

One of the most relevant findings in our study was the variability of the antiproteinuric response according to the treatment group. Response proportions varied descriptively across concomitant treatment categories. This observation is consistent with the increasing evidence that suggests complementary effects between therapeutic strategies and is aligned with the current therapeutic recommendations [17]. However, treatment regimens were selected in routine clinical practice rather than randomly assigned, and baseline comparability among treatment groups was not established. Therefore, these findings may reflect differences in baseline disease severity, clinical indications, treatment tolerance, or other unmeasured factors. They should not be interpreted as evidence that a particular combination independently increased the probability of antiproteinuric response or was more effective than another regimen.

Concerning safety, serum potassium increased from baseline to six months across the response categories. Although a modest increase in the serum potassium levels was observed in the responder groups, values reached remained within clinically manageable ranges and no relevant occurrence of severe hyperkalemia was documented. These findings are consistent with the safety analyses derived from FIDELIO-DKD and the hyperkalemia-specific risk studies, which have shown that appropriate potassium monitoring allows a favorable benefit–risk balance to be maintained for most patients with diabetic kidney disease [19,20].

In the FIDELIO-DKD study, hyperkalemia was more frequent in the group treated with finerenone than in the placebo group; the occurrence of serious events and permanent treatment discontinuation was low [19]. Subsequently, the pooled analysis of the FIDELITY program confirmed that the kidney and cardiovascular benefit of finerenone widely outweighs the risk associated with the increase in serum potassium when adequate monitoring is performed [11]. Also, specific studies about hyperkalemia have shown that most episodes are mild or moderate, transient and manageable through therapeutic adjustments and periodic monitoring, without the need to discontinue treatment in the majority of cases [19,20,21]. The increase in serum potassium observed in this cohort should be interpreted in the context of evidence from randomized trials, in which the risk of clinically relevant hyperkalemia was generally manageable through appropriate patient selection, periodic potassium monitoring, and dose adjustment when required. Therefore, concern about hyperkalemia should not, by itself, preclude the use of finerenone in eligible patients with diabetic kidney disease. Nevertheless, because clinically relevant hyperkalemia events and treatment modifications were not systematically captured in the present study, our findings should not be considered an independent confirmation of its safety profile.

Several limitations should be considered when interpreting these findings. First, the retrospective, uncontrolled, post hoc design precludes causal attribution of the observed UACR changes to finerenone. Patient selection, concomitant kidney-protective therapies, changes in blood pressure or glycemic control, and other measured or unmeasured factors may have influenced the results. In addition, the analysis was restricted to patients with UACR measurements available at baseline and six months, introducing the possibility of complete-case selection bias. Second, UACR at each time point was based on a single measurement. Given its substantial intraindividual biological and analytical variability, random variation and regression to the mean may have influenced both the magnitude of the observed changes and the classification of patients, particularly those close to the 30% and 50% thresholds. Third, the marked imbalance in group sizes, including only 27 non-responders, limited the precision of subgroup analyses. No multivariable analysis was performed; therefore, the study could neither identify nor exclude independent predictors of response. Fourth, the six-month follow-up precluded assessment of long-term kidney and cardiovascular outcomes or determination of whether early UACR changes were associated with subsequent clinical benefit. Finally, clinically relevant hyperkalemia events, finerenone dose changes, treatment interruption, and discontinuation were not systematically captured, limiting the assessment of safety and tolerability.

The study also has relevant strengths, including its multicenter design, the inclusion of patients from seven Latin American countries, and the evaluation of UACR changes after finerenone initiation in routine nephrology practice. These features provide descriptive real-world information that complements, but should not be directly compared with, evidence from randomized controlled trials.

## 5. Conclusions

In this retrospective post hoc analysis of a Latin American cohort, most patients with available baseline and six-month measurements experienced a reduction in UACR following finerenone initiation. These findings describe changes observed in routine clinical practice but do not establish a treatment-specific effect. The absence of a comparator group, concomitant therapies, potential confounding, and reliance on single UACR measurements at each time point preclude attributing the observed reductions exclusively to finerenone. Biological variability and regression to the mean may also have influenced response classification. These exploratory findings warrant further investigation in prospective controlled studies with repeated UACR measurements and prespecified adjusted analyses.

Prospective studies with long-term follow-up are necessary to determine whether the differences observed between responders and non-responders translate into long-term clinical benefits.

## Figures and Tables

**Figure 1:**
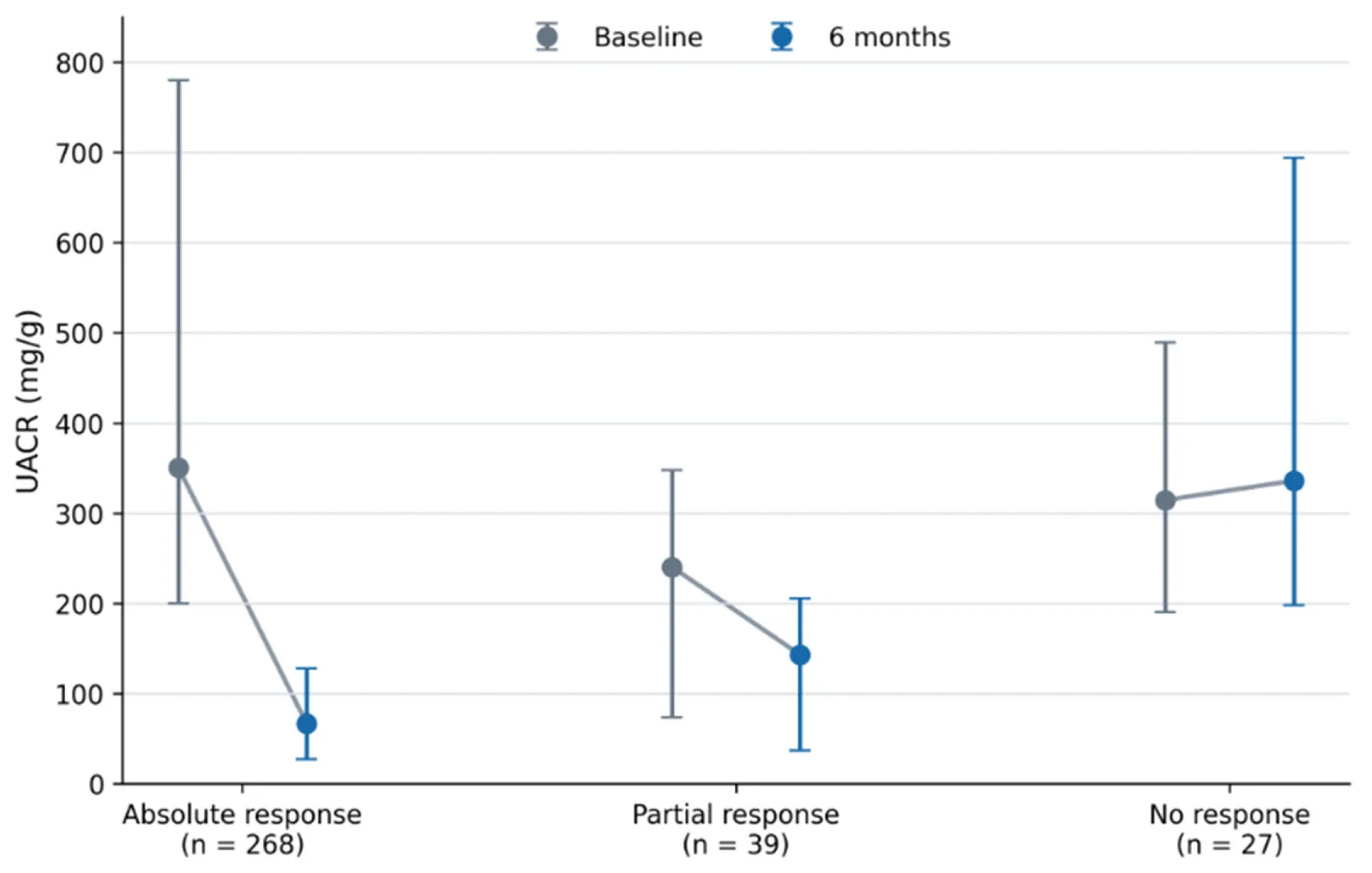
Urinary albumin-to-creatinine ratio at baseline and six months according to response category.

**Table 1:** Clinical and laboratory characteristics at baseline and six months according to UACR response category.

Group	Variable	Baseline (Median-IQR)	6 Months (Median-IQR)	*p*-Value
Absolute responder *n* = 268	Age	63.4 ± 11.8	/	/
	Female gender	45.9%	/	/
	Arterial hypertension	88.6%	/	/
	Atherosclerotic cardiovascular disease	37.1%	/	/
	Smoking	13.1%	/	/
	Acute myocardial infarction	18.1%	/	/
	Obesity	30.4%	/	/
	UACR (mg/g)	350.70 [200–780]	67.00 [27–128]	<0.0001
	eGFR (mL/min/1.73 m^2^)	37.61 [30–48]	40.77 [27.25–51]	0.9714
	Systolic blood pressure (mmHg)	145 [133–160]	130 [120–140]	<0.0001
	Potassium (mEq/L)	4.40 [4.10–4.60]	4.70 [4.38–5]	<0.0001
Partial responder *n* = 39	Age	61.7 ± 11.9	/	/
	Female gender	43.6%	/	/
	Arterial hypertension	82.4%	/	/
	Atherosclerotic cardiovascular disease	44.1%	/	/
	Smoking	11.8%	/	/
	Acute myocardial infarction	14.7%	/	/
	Obesity	29.4%	/	/
	UACR (mg/g)	240.50 [73.66–347.75]	143 [37.31–205.50]	<0.0001
	eGFR (mL/min/1.73 m^2^)	46.15 [40.25–56.00]	48.80 [42.00–56.00]	0.0831
	Systolic blood pressure (mmHg)	135 [130–146.50]	130 [122.50–139.50]	<0.0001
	Potassium (mEq/L)	4.35 [3.90–4.50]	4.40 [4.15–4.75]	0.0045
Non-responder *n* = 27	Age	58.0 ± 10.8	/	/
	Female gender	40.7%	/	/
	Arterial hypertension	66.7%	/	/
	Atherosclerotic cardiovascular disease	33.3%	/	/
	Smoking	20.8%	/	/
	Acute myocardial infarction	20.8%	/	/
	Obesity	41.7%	/	/
	UACR (mg/g)	314.50 [190–489.50]	336 [197.75–693.75]	0.4434
	eGFR (mL/min/1.73 m^2^)	41 [33–53]	37 [20–51]	0.0023
	Systolic blood pressure (mmHg)	147 [120–170]	134 [122.50–152.50]	0.0592
	Potassium (mEq/L)	4.60 [4.43–4.98]	5.15 [4.72–5.58]	0.0006

*n* = number of patients; IQR: interquartile range; UACR: urine albumin-creatinine ratio; eGFR: estimated glomerular filtration rate.

**Table 2:** Albuminuria response according to therapeutic group.

Treatment Category	Absolute Responder (≥50%)	Partial Responder (30–49%)	Non-Responder (<30%)
Finerenone + RAASi + SGLT-2i *n* = 150	75.3% 113	14.7% 22	10% 15
Finerenone + SGLT-2i *n* = 75	98.7% 74	0	1.3% 1
Finerenone + RAASi + GLP-1Ra + SGLT-2i *n* = 16	62.5% 10	18.75% 3	18.75% 3
Others *n* = 93	76.3% 71	15.1% 14	8.6% 8

*n* = number of patients; RAASi: renin–angiotensin–aldosterone system inhibitors; SGLT-2i: sodium-glucose cotransporter 2 inhibitors; GLP-1Ra: glucagon-like peptide-1 receptor agonists.

## Data Availability

The original contributions presented in this study are included in the article. Further inquiries can be directed to the corresponding author.
